# The relationship between nerve conduction velocity and fiber morphology during peripheral nerve regeneration

**DOI:** 10.1002/brb3.61

**Published:** 2012-07

**Authors:** Masayoshi Ikeda, Yoshinori Oka

**Affiliations:** Department of Orthopaedic Surgery, Tokai University Oiso HospitalKanagawa 259–0198, Japan

**Keywords:** Axon diameter, fiber diameter, *g*-ratio, internodal length, motor nerve conduction velocity, nerve regeneration

## Abstract

We analyzed the relationship between motor nerve conduction velocity (MCV) and morphological changes in regenerating nerve fibers at different times after sciatic nerve transection to identify reliable indices of functional recovery. Thirty rats were divided into five equal groups, one control group and four groups subjected to sciatic nerve transection and immediate suturing, followed by regeneration for 50, 100, 150, and 200 days, respectively. MCV was measured in each group, followed by morphometric analyses of fibers of the common peroneal nerve. MCV increased progressively with time after nerve transection, although it remained lower than the control velocity. Mean fiber diameter (axon plus myelin sheath) also increased with time after nerve transection. Recovery of mean fiber diameter was well correlated with MCV, even though regenerating nerves likely contained many small nonconducting fibers. In contrast, the change in the mean diameter of regenerating axons and relative myelin thickness (*g*-ratio) did not provide an accurate measure of recovery as they were not increasing in a time-dependent manner. Furthermore, internodal length changed only slightly with increasing fiber diameter in regenerating nerves; therefore, the regression relation between fiber diameter and internodal length was not a sensitive index of recovery. MCV and mean fiber diameter were the most sensitive indices of functional recovery during sciatic nerve regeneration.

## Introduction

Numerous experimental studies have investigated morphological parameters that may affect conduction velocity in myelinated nerve fibers. These parameters include fiber diameter, axon diameter, myelin thickness, and internodal length (Waxman 1980). Among these parameters, it is clear that conduction velocity is closely related to fiber diameter and myelin thickness. These relations were first proposed on the basis of theoretical considerations (Rushton 1951; Moore et al. 1978) and subsequently confirmed by experimental studies in both intact and regenerating nerve fibers (Gutmann and Sanders 1943; Berry et al. 1944; Sanders and Whitteridge 1946; Cragg and Thomas 1964; Schröder 1972). The conduction velocity is proportional to fiber diameter, and there is an optimum ratio of myelin thickness to fiber diameter for maximal conduction velocity. Internodal length is roughly proportional to fiber diameter in normal nerve populations (Hiscoe 1947; Vizoso and Young 1948; Vizoso 1950). However, this relationship tends to break down during nerve regeneration because internodal lengths remain abnormally short, in contrast to more complete recovery of fiber diameter and myelin thickness (Cragg and Thomas 1964; Beuche and Friede 1985; Hildebrand et al. 1985; Gattuso et al. 1988).

The purpose of this study is to analyze the relationship between motor nerve conduction velocity (MCV) and morphological changes in individual fibers, including fiber diameter, myelin thickness, and internodal length, during regeneration of peripheral nerves. The most reliable indices of regeneration were determined by regression analysis at different time points following sciatic nerve transection. We found that MCV and mean fiber diameter were the most reliable indices of functional recovery during regeneration.

## Materials and Methods

Thirty male Sprague–Dawley rats, weighing approximately 600–700 g, were used for this study, including six control rats and four groups of six rats each subjected to sciatic nerve transection, suturing, and recovery for 50, 100, 150, or 200 days, respectively. Rats in the nerve transection groups were anesthetized by face mask inhalation of 5% halothane. The left sciatic nerve was exposed through a lateral incision in the mid-thigh. The nerve was transected sharply with micro-scissors 2-cm distal to the sciatic notch and immediately repaired with 10–0 nylon epineural sutures (Ethilon, Ethicon Ltd., UK) under a dissection microscope (Wild Heerbrugg Ltd., Switzerland). The muscle and skin were closed with 3–0 absorbable sutures (Vicryl, Ethicon Ltd., UK). Our institutional review board approved this study, and every effort was made to reduce the number of animals used and their suffering.

At 50, 100, 150, and 200 days after initial surgery, the rats were anesthetized and the sciatic nerve was exposed from the trochanteric notch to the common peroneal nerve. MCV was measured as described below. Rats were then sacrificed and the common peroneal nerve was removed for morphological analysis.

### Motor nerve conduction study

The rat was wrapped in a bubble packing sheet and aluminum foil to maintain body temperature above 37°C. A Medelec Sapphire II electromyography unit (Medelec Ltd., UK) was used for stimulation and recording of compound motor action potentials (CMAPs). Two bipolar electrodes were used for stimulation; one was placed proximally on the sciatic nerve near the obturator foramen and the other on the common peroneal nerve just distal to the division; this way, the distance between the electrodes was maximum. CMAPs were recorded from the extensor digitorum longus using two 6-mm recording disc electrodes; the active electrode was applied to the muscle belly through the skin and the reference electrode was applied to the muscle tendon. The nerve was stimulated by a supramaximal 50 μsec constant current.

MCV was calculated by the conventional method: MCV (m/sec) =*L*/*T*, where *L* (m) is the distance between the two stimulus electrodes and *T* (sec) is the difference in delay between CMAPs evoked by the proximal and distal stimulating electrodes.

### Morphometric analysis

After completion of the motor nerve conduction studies, a 15-mm segment of the common peroneal nerve from the sciatic nerve bifurcation to the muscular insertion was excised. The proximal one-third of the segment was processed for preparation of semi-thin transverse slices (1 μm). Briefly, the nerve segment was fixed in 2.5% cacodylate buffered glutaraldehyde (pH, 7.3) at 4°C for 1 h and cut into 1-mm transverse sections. Sections were placed in the same fixative solution for an additional 12 h, postfixed in 1% cacodylate buffered OsO_4_ for 2 h, dehydrated, and embedded in Araldite. Semi-thin sections were prepared and stained with toluidine blue. These slices were used for the measurement of fiber and axon diameters.

For measurement of internodal length, the distal two-thirds of the segment was fixed in 8% cacodylate buffered formalin (pH 7.2) for 48 h and fixed in 1% cacodylate buffered OsO_4_ for 24 h. After washing in distilled water, the nerve was mounted in 50% glycerol solution under a stereomicroscope (Wild Heerbrugg Ltd., Switzerland), and the individual fibers were gently teased apart using a fine needle.

**Figure 1:**
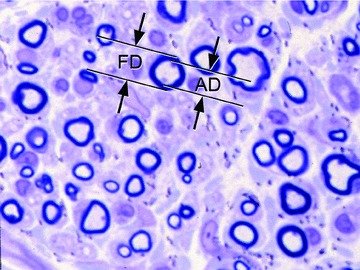
Semi-thin section of a sciatic nerve. Cross-section of a sciatic nerve 200 days after transection and repair. The shortest lengths of the outer and inner margins of the myelin sheath were measured to determine the fiber diameter (FD) and axon diameter (AD). Magnification is 1000×.

**Figure 2:**
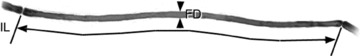
Teased nerve fiber. Teased nerve segment 200 days after transection and repair. The internodal length between the nodes of Ranvier (IL) and the fiber diameter (FD) were measured. Magnification is 400×.

Morphometric measurements were performed using a VIDS III image analysis system (Analytical Measuring System Ltd., UK) connected to a microscope. The image was viewed and digitized on a display screen, and the morpho-logical parameters were measured using a cursor and digitizing table (Fig. 1). The data were saved on an IBM XT computer (IBM Corporation, USA) interfaced to the VIDS III system. Fiber and axon diameters of the semi-thin sections were measured at 1000× magnification in five different fields for each specimen (rat), following which they were stored and summarized for statistical analysis. Fiber diameter and internodal distance of the teased nerve segments were measured at 400× magnification in 300 fibers of each specimen (rat), following which they were stored and summarized for statistical analysis (Fig. 2).

### Statistical analysis

MVC was compared between groups using a nonparametric Kruskal–Wallis one-way analysis of variance by ranks test. Morphometric parameters were expressed as mean ± standard error of the mean, and pairwise differences were tested using Tukey's honestly significant difference and Student's *t*-tests. Relationships between the morphometric parameters and time after nerve transection were tested by best fit analyses using linear or logarithmic equations, and the correlation coefficients (*r*) were tested. *P* < 0.05 was considered statistically significant.

## Results

### Motor nerve conduction study

The mean MCV in the control group was 74.2 m/sec; this was significantly higher than that measured in the four rat groups allowed to recover for 50, 100, 150, and 200 days (Table 1). The mean MCV increased with time between 50 and 200 days after transection, and the mean MCV of the 200-day regeneration group was significantly higher than that of the 50-day group, indicating partial regeneration and functional recovery of the sciatic and common peroneal nerves. There was no significant difference between the 100-, 150-, and 200-day groups.

**Table 1:** Motor nerve conduction velocities and morphometric measurements at different times after nerve transection

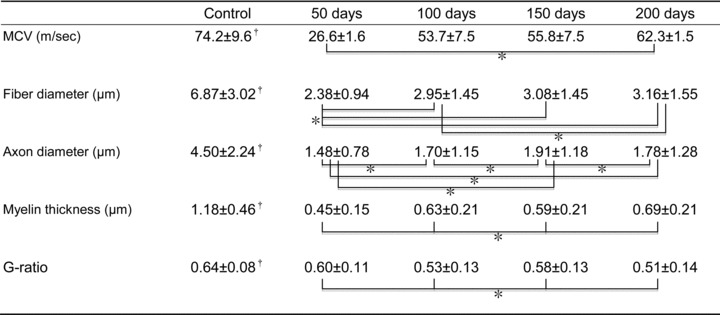

Values are expressed as mean ± standard deviation. A significant difference between mean values at different times after transection is indicated by *P < 0.05. † denotes that the control group values were significantly larger than the transection group values. For myelin thickness and *g*-ratio, there were significant differences between all posttransection groups.

### Morphometric analysis

**Figure 3:**
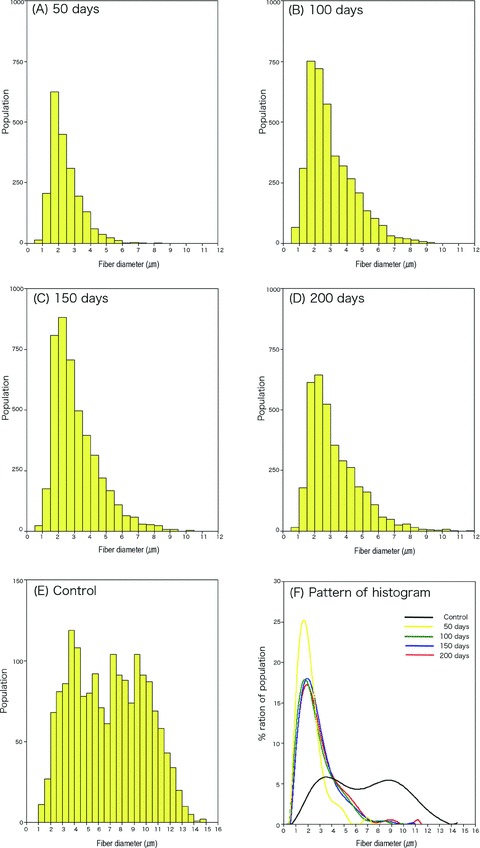
Histograms of fiber diameter. The distribution of nerve fiber diameters was unimodal at 50 days (*n*= 2065) (A), 100 days (*n*= 3993) (B), 150 days (*n*= 4520) (C), and 200 days (*n*= 3532) (D) after transection of the sciatic nerve. In contrast, the fiber diameter histogram of the control nerve was bimodal with peaks at 4.5 and 9.5 μm (*n*= 1763) (E). Each group included five rats and whole nerve fivers from five different consecutive fields were measured and stored for each nerve specimen. Distribution patterns at different time points and control nerve were combined and superimposed. Lines are drawn using different colors: control with black, 50 days with yellow, 100 days with green, 150 days with blue, and 200 days with red (F). The proportion of the larger nerve fibers increased with time between 50 and 200 days after transection.

Figure 3 presents the frequency distribution histograms of fiber diameter as measured in the control and transection groups. All four histograms compiled from the common peroneal nerves studied after 50, 100, 150, and 200 days of recovery were unimodal, with the proportion of nerve fibers shifted to larger nerve diameters (to the right) with longer recovery times (Fig. 3A–D). In contrast, fiber diameters exhibited a bimodal distribution in the normal sciatic nerves, with main peaks at 4.5 and 9.5 μm (Fig. 3E).

In all nerve transection groups, both mean fiber diameter and axon diameter were significantly smaller than those in the control group. Mean fiber diameter increased with time between 50 and 200 days after transection. The mean fiber diameter of the 50-day regeneration group was significantly smaller than that measured in the other groups (Table 1). The mean fiber diameter of the 200-day group was significantly larger than that of the 100-day group. In contrast to the time-dependent increase in mean fiber diameter, the mean axon diameter was significantly larger in the 150-day group than in the other groups (followed in order by the 200-, 100-, and 50-day groups). The mean myelin thickness of the regenerated nerve fibers was largest in the 200-day group, followed in descending order by the 100-, 150-, and 50-day groups; there were significant differences between each group. There were also significant differences in mean *g*-ratio (quotient axon diameter/fiber diameter, a measure of relative myelin thickness) between each group. The mean *g*-ratio of the control group was larger than that of any transection group. The mean value of the *g*-ratio was highest in the 50-day group, followed in descending order by the 150-, 100-, and 200-day groups. Therefore, only MCV and mean fiber diameter demonstrated a consistent relationship with recovery time.

**Figure 4:**
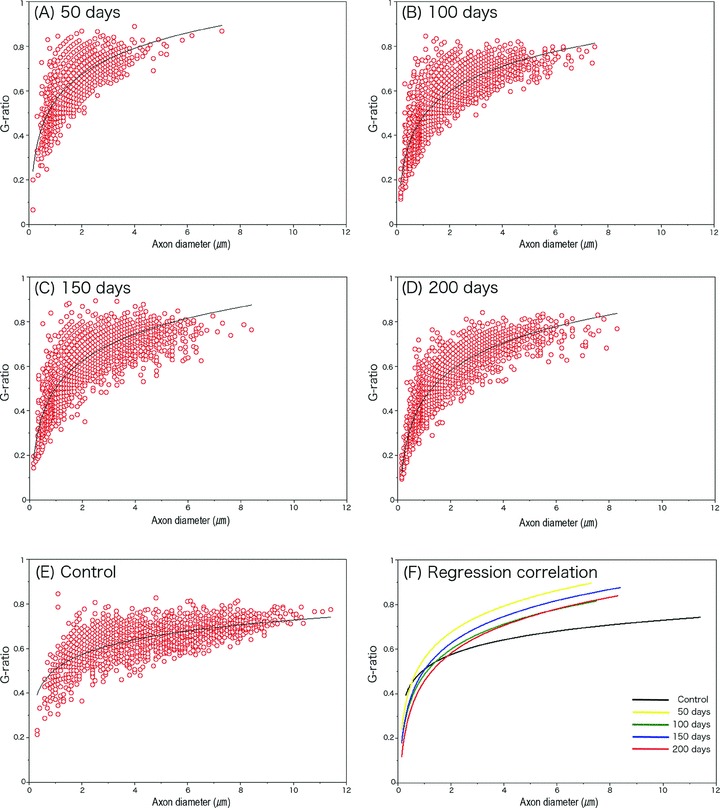
Scatter plots of axon diameter against *g*-ratio (axon diameter/fiber diameter). The axon diameter versus *g*-ratio relation at 50 days (*n*= 2065) (A), 100 days (*n*= 3993) (B), 150 days (*n*= 4520) (C), and 200 days (*n*= 3532) (D) in the transection group. Each group included five rats and the whole nerve fivers from five different consecutive fields were measured and stored for each nerve specimen. The axon diameter versus *g*-ratio relation in the control group (*n*= 1763) (E). The *g*-ratio of each measured fiber is indicated by a single red circle (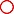). Note that red circles to the lower left represent small diameter axons with relatively thick myelin sheaths (low *g*-ratio) that are likely to be nonconducting. Correlation between axon diameter (*x*_1_) and *g*-ratio (*y*_1_) is expressed by the correlation coefficient (*r*_1_) of the logarithmic regression curve. (A) *y*_1_= 0.393log(*x*_1_) + 0.555 (*r*_1_= 0.735). (B) *y*_1_= 0.379log(*x*_1_) + 0.482 (*r*_1_= 0.861). (C) *y*_1_= 0.402log(*x*_1_) + 0.503 (*r*_1_= 0.839). (D) *y*_1_= 0.419log(*x*_1_) + 0.453 (*r*_1_= 0.910). (E) *y*_1_= 0.220log(*x*_1_) + 0.508 (*r*_1_= 0.702). (F) Logarithmic regression curves of axon diameter against *g*-ratio for the control (black) and regenerated nerve fibers (50 days, yellow; 100 days, green; 150 days, blue; 200 days, red). Lines are nearly superimposed, indicating that this relation is a poor index of recovery.

Scatter plots of axon diameter against *g*-ratio revealed a significant correlation within each group (Fig. 4). At each time point after transection, the *g*-ratio to axon diameter relation was best fit by the following logarithmic equation:


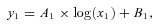


where *x*_1_ is the axon diameter and *y*_1_ is the *g*-ratio.

The correlation coefficients (*r*_1_) ranged from 0.735 to 0.910. Time after transection was associated with a rightward shift in these plots, indicating more numerous axons with large diameters and higher *g*-ratios. In each transection group, axon diameters were as large as 9 μm. At 50 days following transection, however, most axons where smaller than 6 μm, and the *g*-ratio increased steeply with increasing diameter (Fig. 4A). By 150–200 days (Fig. 4C and D), the curve extended to 9 μm. The tail region at the lower left side of the plots indicates the presence of very thin fibers with excessively thick myelin sheaths (low *g*-ratio). Many axons in the 150- and 200-day groups exhibited these characteristics, while few such axons were found in the control sciatic nerves (Fig. 4E).

**Figure 5:**
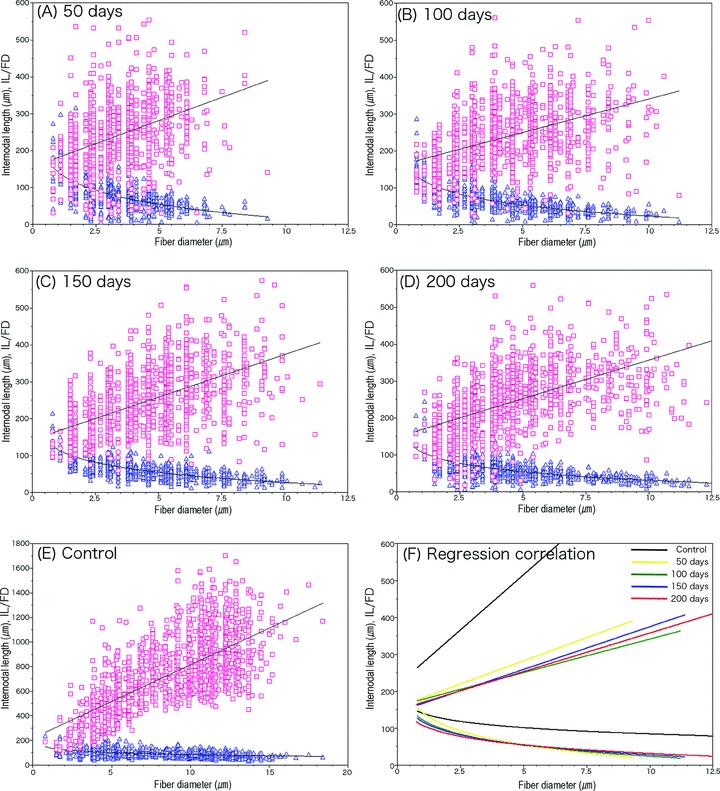
Scatter plots of fiber diameter against internodal length and quotient internodal length/fiber diameter (IL/FD). Fifty days (*n*= 1146) (A), 100 days (*n*= 1421) (B), 150 days (*n*= 1432) (C), and 200 days (*n*= 1452) (D) after nerve transection. Control values (*n*= 1333) (E). Each group included five rats, and about 300 nerve fibers from each specimen were measured. The internodal length of each measured fiber is denoted by (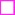), while IL/FD for each fiber is represented by (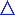). Correlations between fiber diameter (*x*_2_) and internodal length (*y*_2_) at each time point after nerve transection are expressed as linear regression lines, and the correlations between fiber diameter (*x*_3_) and quotient IL/FD (*y*_3_) are expressed as logarithmic regression curves with correlation coefficients *r*_2_ and *r*_3_. (A) *y*_2_= 25.105*x*_2_+ 156.426 (*r*_2_= 0.393), *y*_3_=−126.192log(*x*_3_) + 142.199 (*r*_3_= 0.628). (B) *y*_2_= 18.171*x*_2_+ 158.890 (*r*_2_= 0.450), *y*_3_=−99.680log(*x*_3_) + 122.398 (*r*_3_= 0.745). (C) *y*_2_= 23.125*x*_2_+ 142.318 (*r*_2_= 0.542), *y*_3_=−90.294log(*x*_3_) + 116.988 (*r*_3_= 0.717). (D) *y*_2_= 20.943*x*_2_+ 147.218 (*r*_2_= 0.548), *y*_3_=−76.580log(*x*_3_) + 107.227 (*r*_3_= 0.647). (E) *y*_2_= 59.969*x*_2_+ 215.296 (*r*_2_= 0.694), *y*_3_=−56. 502log(*x*_3_) + 139.792 (*r*_3_= 0.384). (F) Linear regression lines of fiber diameter against internodal length and logarithmic regression curves of fiber diameter against IL/FD of the regenerated nerve fibers are superimposed, indicating that these relations are not sensitive indices of regeneration. Lines are plotted using different colors: control with black, 50 days with yellow, 100 days with green, 150 days with blue, and 200 days with red.

We then investigated the quantitative relationship between fiber diameter and internodal length (Fig. 5). Following nerve transection, internodal length varied considerably among fibers, as evidenced by the higher scatter of diameter versus internodal length points (Fig. 5A–D), although it was directly related to fiber diameter in normal nerves (Fig. 5E). The internodal length of most regenerated fibers ranged from 100 to 400 μm, and it tended to increase with increasing fiber diameter at each stage when the fiber diameter was smaller than around 7 μm. However, when the fiber diameter exceeded 7 μm after 100 days, the internodal length tended to decrease with increasing fiber diameter. The correlation between fiber diameter (*x*_2_) and internodal length (*y*_2_) was expressed as a linear regression line by the following formula: *y*_2_=*A*_2_×*x*_2_+*B*_2_, where *x*_2_ is fiber diameter and *y*_2_ is internodal length. As shown in Figure 3, the linear regression lines for the transected nerves at 50, 100, 150, and 200 days have significantly flatter slopes than those for the control nerves. Furthermore, the correlation between fiber diameter and internodal length was weaker at every posttransection time point (coefficients ranging from 0.393 to 0.694). In contrast, there was a strong correlation between the fiber diameter (*x*_3_) and the ratio of the quotient internodal length to fiber diameter (IL/FD) (*y*_3_), with a logarithmic regression curve as follows: *y*_3_=*E*× log(*x*_3_) +*F.* Indeed, although the logarithmic relation in normal nerves was weak (*r*_3_= 0.384), there were strong logarithmic correlations at each time point during nerve regeneration (coefficients ranging from 0.628 to 0.745). When the regression curves for regenerating fibers were superimposed, the regression curves at each time point after transection were overlapping, indicating that this fiber diameter to IL/FD relationship is also a poor index of functional recovery.

## Discussion

New nerve repair techniques should only be introduced into general clinical practice if they can be conclusively proved efficient in improving the results obtained from previous techniques. To reach this goal, evaluation methods that provide an objective measure of recovery are required. Animal models also provide objective measures of functional recovery in a manner not presently obtainable in clinical studies. Morphological and electrophysiological measures reflect the inherent variability in the rate of nerve regeneration, myelination, and functional recovery; therefore, a combination of electrophysiological and morphometric measures may yield the best indication of recovery, especially over multiple time points. We demonstrated that recovery of MCV and mean fiber diameter were well correlated with time after sciatic nerve transection. Although mean myelin thickness, axonal diameter, and *g*-ratio decreased after transection, they were not well correlated with time or MCV recovery.

Conventional MCV measurements tend to reflect primarily upon the faster conducting fibers and provide little information about the conduction properties of the entire population of regenerating fibers (Rosen and Jewett 1980; Dorfman 1984). The present study showed that MCV progressively increased through 50–200 days after transection, although it did not return to normal by 200 days. These observations reflect the recovery process of the regenerated fibers. Conduction velocity increases in appropriate proportion to fiber diameter (Rushton 1951; Moore et al. 1978); therefore, the increase in MCV should reflect an increase in the relative number of fibers with large diameters. Indeed, the histograms plotted in our study revealed a substantial increase in the number of fibers with large diameters during recovery. While peak posttransection MCV was within 80% of that measured in intact nerves, mean fiber diameter remained substantially below that of the intact nerves. Moreover, the histograms for fiber diameter in the transection group revealed a unimodal distribution at all time points up to 200 days, while the fiber diameter distribution for the control group was bimodal, with a significantly higher proportion of fibers with large diameters. Dissociation between MCV recovery and mean fiber diameter recovery, which was calculated from the whole fibers, is therefore expected. This may simply imply that many nonfunctional regenerating fibers could not be eliminated morphologically, or that there were no significant differences in MCV between the various groups.

Many of the fibers with small diameters may in fact be nonconducting and degenerating. As the nerve fibers regenerate distally and reach the appropriate target organ, fiber diameter increases and the myelin sheath grows (Weiss et al. 1945; Schröder 1972; Myles and Glasby 1991). If sprouting axons do not make an appropriate connection with the target organ, they are denied vital growth factors and degenerate. It has been demonstrated that in rat sciatic nerves, there is an initial increase in the number of fibers distal to the site of transection, followed by a gradual decrease (Mackinnon et al. 1991). The initial increase can last for approximately six months before axonal number slowly decreases back to pretransection levels over the following two years. It may be difficult to distinguish smaller, successfully regenerated fibers from atrophic, dying fibers, especially during the early phase of regeneration. Therefore, if studies on the morphological evaluation of rat sciatic nerves are completed within six months, their results may be considered inappropriate.

There was a marked dissociation between axon diameter and myelin thickness during regeneration (Cragg and Thomas 1964; Schröder 1972). Regenerated fibers have thinner myelin sheaths than those of normal fibers, although axonal diameters may approach normal values. In the present study, mean fiber diameters increased with time, and they increased to 46% of the normal value at 200 days after nerve repair; however, mean myelin thickness decreased at 150 days. There is an optimal myelin thickness relative to fiber diameter (as measured by the *g*-ratio) to maximize conduction velocity (Rushton 1951). The scatter plots of *g*-ratio against axon diameter and their regression curves showed that larger fibers had higher *g*-ratios, whereas smaller fibers had excessively low *g*-ratios. The mean axon diameter increased between 50 and 150 days; however, it decreased at 200 days. In contrast, the number of fibers with low *g*-ratios increased at 200 days. The highest number of small-caliber axons with much thicker myelin sheaths (low *g*-ratio fibers) were observed at 100 and 200 days after nerve transection. These fibers with low *g*-ratios may be those that failed to reach their target organ, with ensuing collapse of the myelin sheath around a shrinking axon (Beuche and Friede 1985). Therefore, neither mean axon diameter nor myelin thickness provided an accurate morphological index of recovery because of the prevalence of thin, nonfunctional fibers with relatively thick sheaths in the regenerating nerves.

Historically, internodal length has been regarded as an important determinant of MCV (Waxman 1980). Internodal length is also roughly proportional to fiber diameter in normal fibers (Hiscoe 1947; Vizoso 1950). On the other hand, regenerating fibers have shorter internodes relative to normal fibers of the same diameter, and the regression line for the relationship between internodal length and fiber diameter is represented by a flatter slope (Vizoso and Young 1948; Cragg and Thomas 1964; Friede and Beuche 1985; Guttuso et al. 1988). These observations are consistent with our data. The internodal length in the regenerated fibers remained at around 300 μm, although fiber diameter increased with time. This indicates that internodal length does not increase as significantly as does diameter in regenerating fibers, and the decrease in the internodal length of regenerated fibers is not considered to alter MCV significantly. Hence, the slope of the regression lines for intermodal length between 50 and 200 days may not be considered as a sensitive morphological index of recovery in regenerated fibers.

The relationship between internodal length and MCV exhibited a peak conduction velocity over a broad quotient IL/FD range (between 100 and 200) (Brill et al. 1977). This quotient is thought to maximize the MCV. In the present study, the regression curves of IL/FD against fiber diameter showed a similar trend at all four posttransection time points. Thus, it appears reasonable to assume that this function may be an appropriate relation to maximize the MCV in regenerating fibers. Therefore, the relationship between fiber diameter and internodal length is not a sensitive recovery index.

Thus, we concluded that MCV and mean fiber diameter were the most reliable indices of functional recovery during sciatic nerve regeneration. Furthermore, the regression relation between fiber diameter and internodal length was not a sensitive index of recovery.
